# Imaging the top of the Earth’s inner core: a present-day flow model

**DOI:** 10.1038/s41598-024-59520-7

**Published:** 2024-04-18

**Authors:** Hrvoje Tkalčić, Anatoly B. Belonoshko, Jack B. Muir, Maurizio Mattesini, Louis Moresi, Lauren Waszek

**Affiliations:** 1 Research School of Earth Sciences, The Australian National University, Canberra, ACT 2601 Australia; 2 School of Earth Sciences and Engineering, Nanjing University, Nanjing, 210093 China; 3 Department of Earth Sciences, University of Oxford, Oxford, OX1 3AN UK; 4 Department of Earth’s Physics and Astrophysics, Complutense University of Madrid, Madrid, Spain; 5 Facultad de Ciencias Físicas, Instituto de Geociencias (UCM-CSIC), Madrid, Spain; 6 Physical Sciences, James Cook University, Townsville, QLD 4810 Australia; 7 Department of Physics, New Mexico State University, Las Cruces, NM 88003 Australia

**Keywords:** Geophysics, Solid Earth sciences, Geodynamics

## Abstract

Despite considerable progress in seismology, mineral physics, geodynamics, paleomagnetism, and mathematical geophysics, Earth’s inner core structure and evolution remain enigmatic. One of the most significant issues is its thermal history and the current thermal state. Several hypotheses involving a thermally-convecting inner core have been proposed: a simple, high-viscosity, translational mode, or a classical, lower-viscosity, plume-style convection. Here, we use state-of-the-art seismic imaging to probe the outermost shell of the inner core for its isotropic compressional speed and compare it with recently developed attenuation maps. The pattern emerging in the resulting tomograms is interpreted with recent data on the viscosity of iron as the inner core surface manifestation of a thermally-driven flow, with a positive correlation among compressional speed and attenuation and temperature. Although the outer-core convection controls the heat flux across the inner core boundary, the internally driven inner-core convection is a plausible model that explains a range of observations for the inner core, including distinct anisotropy in the innermost inner core.

## Introduction

Since its discovery in 1936^1^, the Earth’s inner core (IC) has always inspired geoscientists, particularly the disciplines concerning understanding our planet’s internal structure and evolution. From a relatively simple early model of the IC, attributing our planet’s innermost sphere to a phase change of iron at high pressures and temperatures^2^, there emerged a view of the IC playing an active role in the outer-core convection maintaining the geodynamo^3^, a connection with the lowermost mantle^4^ and even with processes at the Earth’s surface^5^. Elastic anisotropy was first proposed to explain anomalous seismological travel times^6^ and normal mode observations^7^, but there is no consensus on its strength or configuration^8^. The IC’s solidity is well-established^9^, although recent observation of IC shear waves in the Earth’s correlation wavefield^10^ revealed a smaller shear modulus than predicted by PREM^11^.

Estimates of many IC properties remain amongst those with the most sizable uncertainty in modern science. For example, studies have found melting temperatures of iron below 5000 and above 7000 K, depending on the method and assumptions about the presence of impurities in the core^12–14^. Similarly, estimates of IC viscosity range from about 10^0^ to 10^22^ Pa·s^15–20^. Moreover, the mineralogical phase at which iron stabilizes at the IC conditions is disputed between the hexagonal closed packed (hcp)^21,22^ and the body center cubic (bcc) phase^23–26^. Recent estimates of thermal conductivity differ^27–29^, but lower values suggest thermal convection in the IC as a mechanism of heat loss. Several hypotheses involving a thermally-convecting inner core have been proposed: classical, lower-viscosity, plume-style^30–32^ convection, and a structurally simpler, high-viscosity, translational mode^33–35^ as a mechanism to explain the hemispherical structure^33,34^. However, new seismological studies of the outermost IC reveal complexities beyond hemispherical isotropic velocity^36–38^ and attenuation^39–41^. Furthermore, the configuration of anisotropy in a radial sense^42^ and differences in results^43,44^ point to more significant structural heterogeneity than previously thought.

## Results

**Figure 1:**
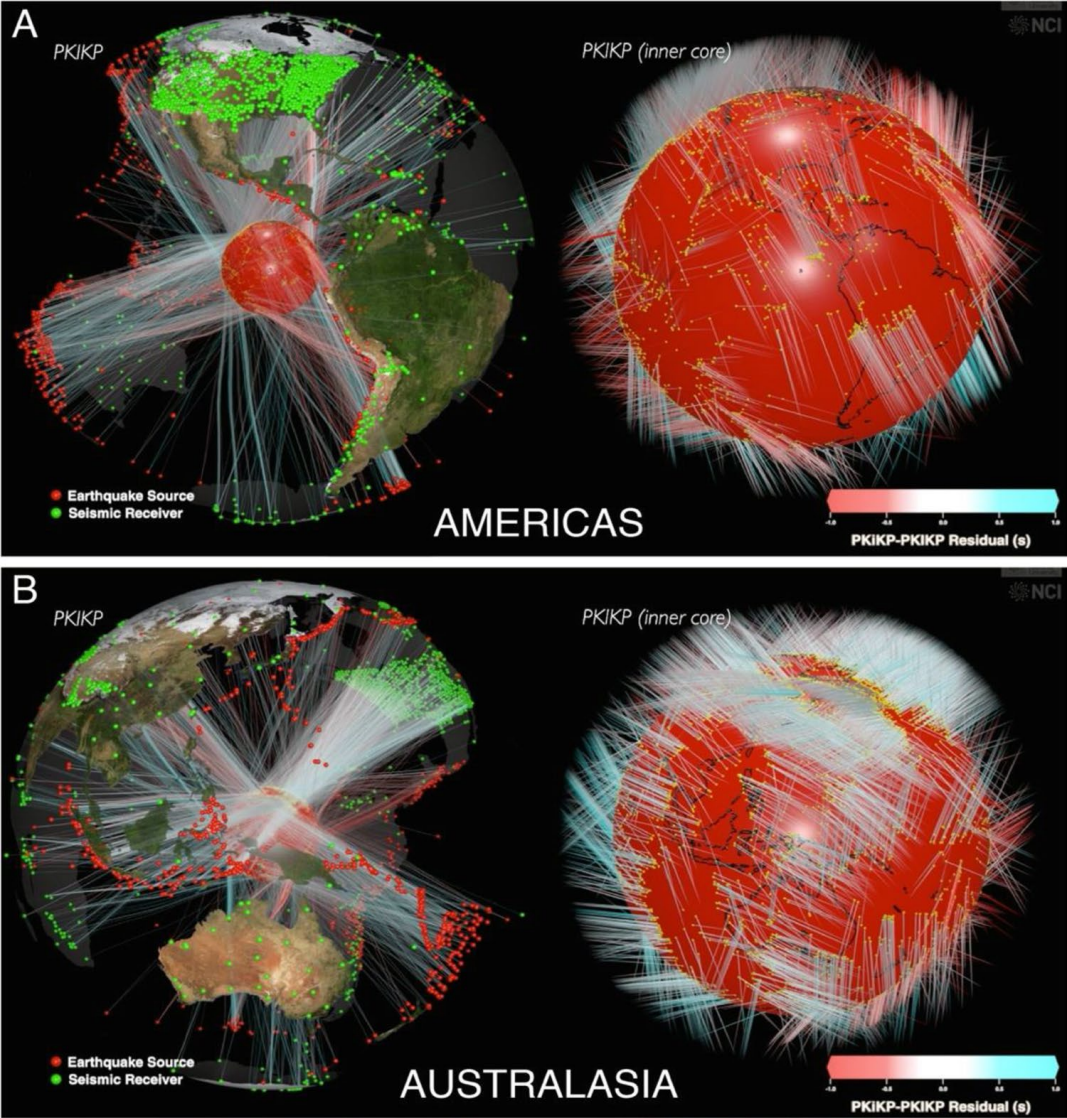
The Earth’s inner core (IC) sampled by the PKIKP seismic waves for two different regions. PKIKP ray-path sampling of the inner core used in this study is illustrated for (**A**) Americas in the quasi-western and (**B**) Australasia in the quasi-eastern hemisphere. Transparent Earth’s interior is on the left and the enlarged IC (red) on the right. Red dots are earthquakes, and green dots are seismic receivers. Rays approximate PKIKP waves’ paths, and their color is proportional to the PKiKP-PKIKP travel time residual relative to the mean. For a 2D illustration of IC sampling, see Fig. S1. For a supplementary 3D animation, see Movie S1.

Motivated by the controversies described above, we exploit recent advances in seismic tomography to probe the outermost 100 km shell of the IC for its isotropic compressional-wave speed. We employ the Hamiltonian Monte Carlo algorithm^45^ for imaging the IC’s top layer using previous^46^ and newly collected PKiKP and PKIKP differential travel times (see section “Methods”). Our scheme utilizes a rigorous treatment of uncertainty applied to data with relatively sparse volumetric sampling, especially in the southern hemisphere (Fig. 1).

**Figure 2:**
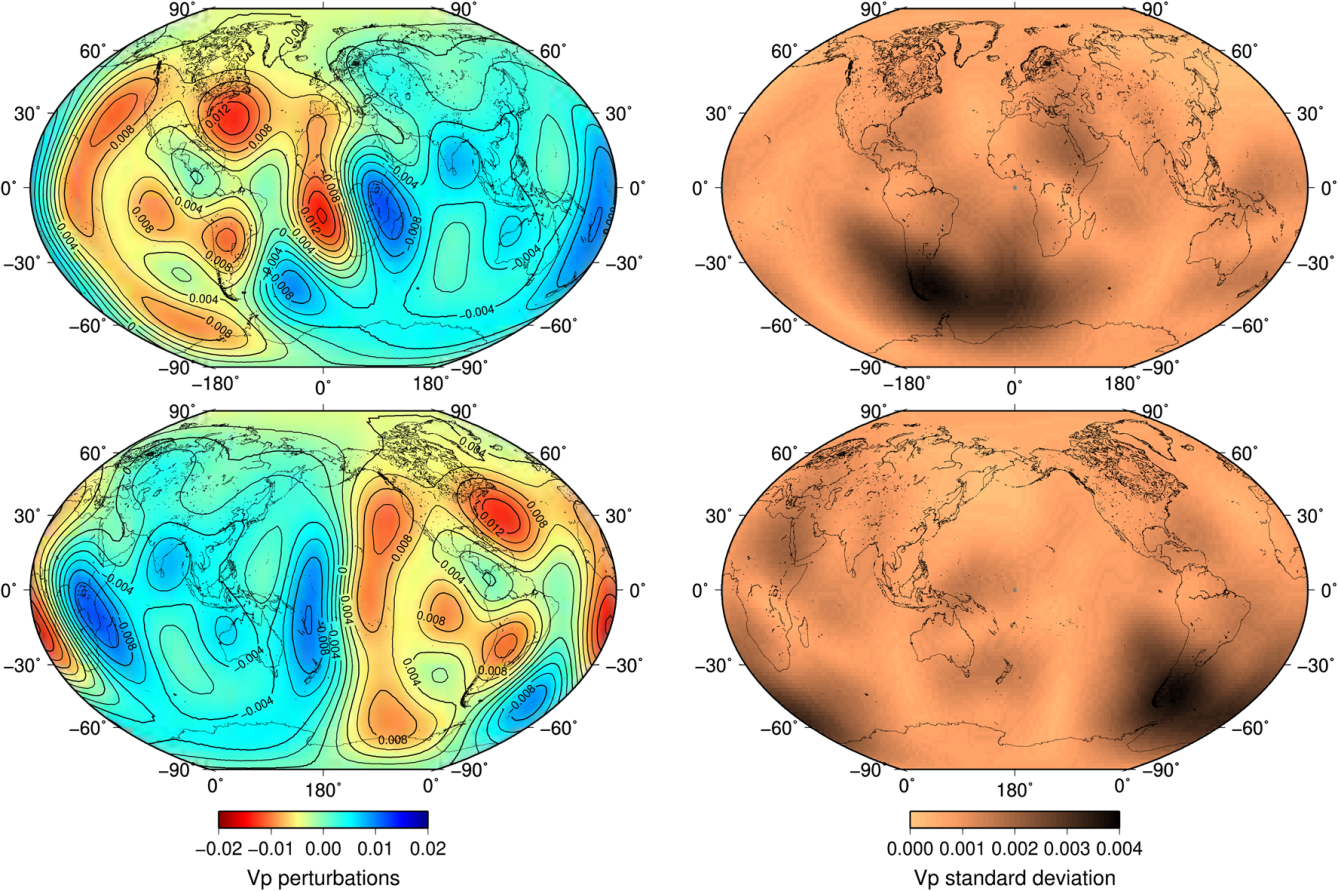
Tomograms of the compressional speed and uncertainty in the outermost IC. Left column: compressional-velocity-perturbation mean with contours centered on 0° (top) and 180° (bottom). Right column: compressional-velocity-perturbation standard deviation centered on 0° (top) and 180° (bottom). For the same tomograms, shown in different projections for more informative viewing, see Figs. S2 and S3.

**Figure 3:**
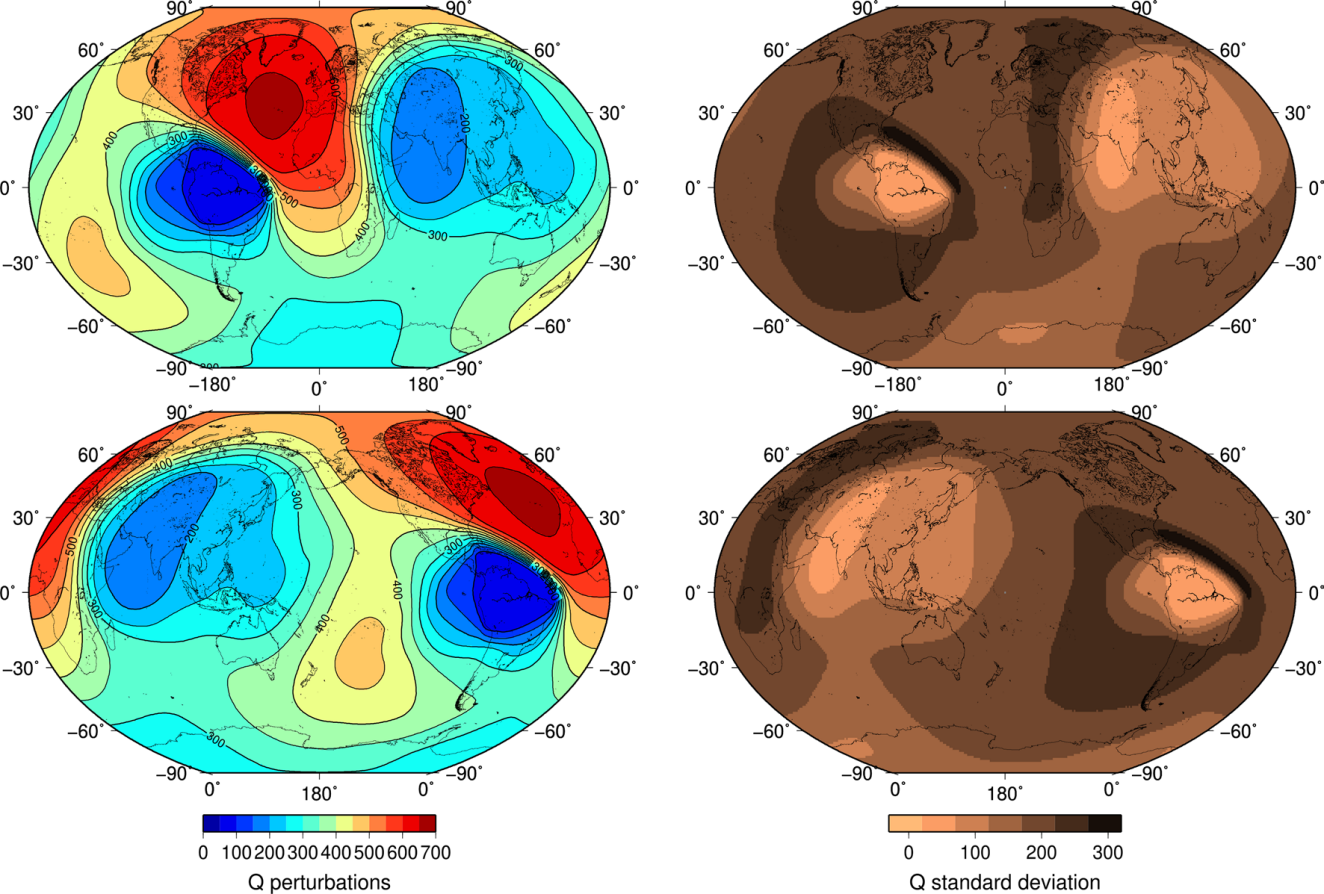
Tomograms of the attenuation quality factor, Q, and its uncertainty in the outermost IC.^9^ Left column: attenuation-quality-factor mean with contours centered on 0° (top) and 180° (bottom). Right column: attenuation-quality-factor standard deviation centered on 0° (top) and 180° (bottom). For the same tomograms, shown in different projections for more informative viewing, see Figs. S4 and S5.

**Figure 4:**
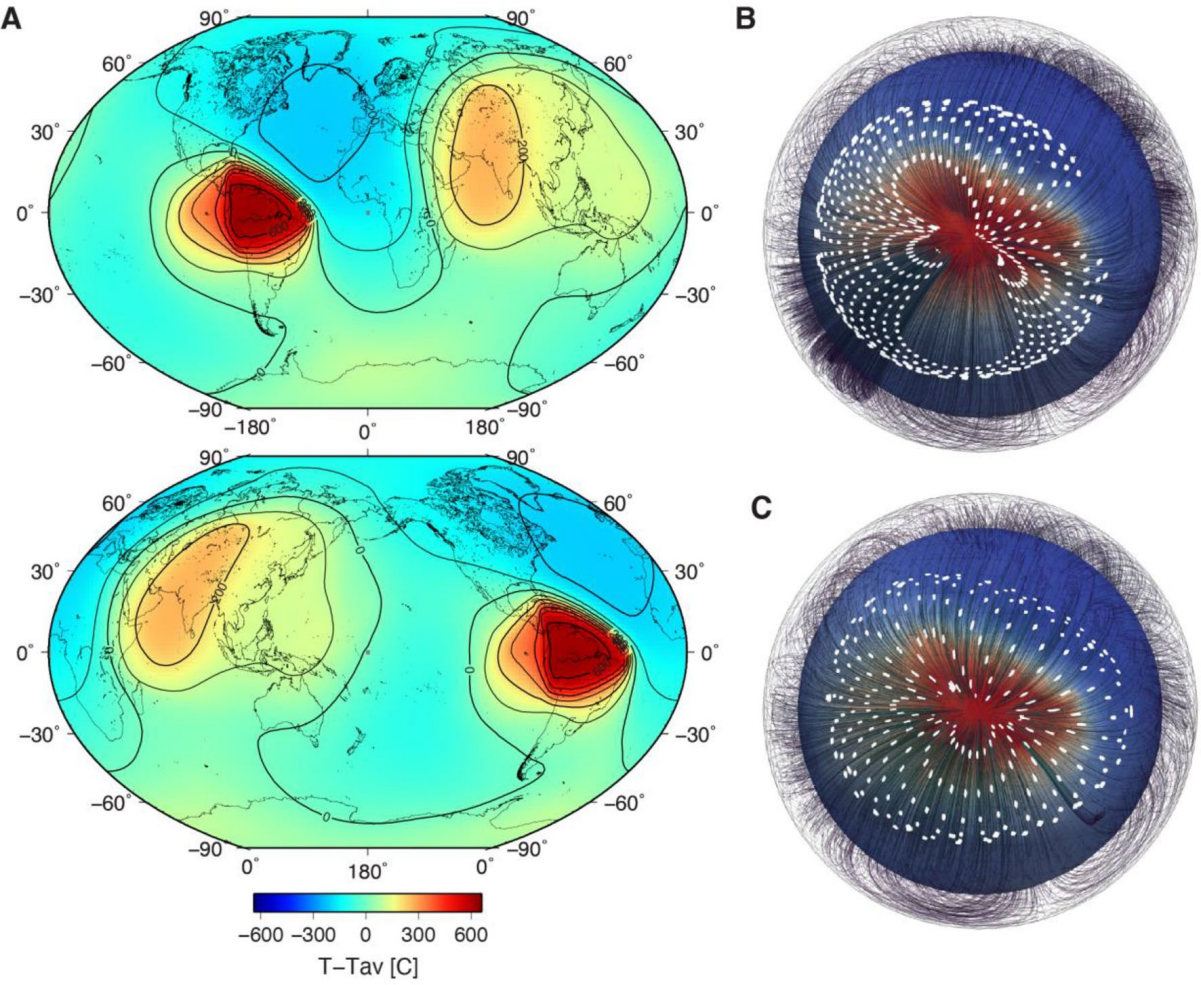
Maps of the temperature field in the outermost IC with the contours and flow lines. (**A**) The maps are centered on 0° (top) and 180° (bottom). For a description of the temperature’s derivation from Q, see section “Methods”. Please note that the temperatures in this map range from − 200 to 600 °C from the average surface temperature for the extreme values of Q. The same maps, but shown in different projections, are in Fig. S9. (**B**) Flow lines for Model 1 (see Table S1) in the outermost 15% of the IC in the domain driven by the density pattern of the tomographic model shown in the red (high temperature)/blue (low temperature) colors on the depth section (see also Fig. S18). Strain markers are introduced at a depth $r=0.3$ and traced over a half-overturn as they spread across the surface. (**C**) As (**B**) but for model 2 which introduces a stagnant innermost inner core layer. The initial depth of the strain markers in this model is $r=0.55$.

We obtain a new tomogram of the isotropic velocity in IC’s outermost 100 km, accompanied by uncertainty (Fig. 2). This result is paired with an attenuation quality-factor tomogram of the IC’s top 400 km from an independent PKIKP and PKPbc wave dataset^41^ (Fig. 3; see section “Methods”). The tomograms reveal the two most striking features in the outermost IC: (1) a more complex pattern of anomalies than a simple, hemispherical configuration, and (2) a positive correlation between isotropic velocity and attenuation on the global scale for the overlapping depths. We suggest that an explanation could be ongoing thermal convection in the IC and that our maps are proxies for density variations in the upper boundary layer of IC convection. From attenuation, we estimate the IC near-surface temperature map (Fig. 4).

While higher values in the compressional-wave speed tomogram (Fig. 2) characterize the IC eastern hemisphere on average, the western hemisphere displays more short-scale variation. Although it is predominantly slower than average, there is a protrusion with higher speed extending from the central Atlantic into Central America, in the region of good recovery with a relatively small standard deviation. The tomogram’s sharpest contrasts exist under the southern part of Africa, striking approximately in the NNW-SSE direction and the central Pacific, bearing N-S. Similarly, localized strong contrasts are also detected in the attenuation tomogram; its apparent slightly lower resolution is attributed to fewer data and averaging over a more extensive depth range.

Higher spherical harmonics present in the expansion of the compressional-wave speed (Fig. 2) and attenuation quality-factor tomograms (Fig. 3) reveal that the IC’s surface in both cases cannot be adequately described by the spherical harmonic degree 1 (hemispherical dichotomy). Our tomography method’s ability to recover complexity from available data is confirmed via synthetic tests (see section “Methods”; Figs. S6–S8, S18). The current volumetric coverage limits the method’s resolution to about the size of the ellipsoidal areas in the synthetic test shown in Fig. S7. Our tomograms contain substantially different features from the lowermost mantle images. This could be due to compositional variations frozen into the IC due to the structure of outer core convection or the lattice-preferred orientation (LPO). The most likely explanation for this deviation from spherical harmonic degree 1 is that the tomograms’ features correspond to the upper parts of ICs convective cells.

There are three distinct areas in the inferred IC convection cell: upward flow associated with hot material rising, horizontal flow along the IC surface (temperature changes from hot to cold), and downward return flow, where cooler material sinks. We estimate the IC temperature via our attenuation model to explore this scenario. We first devise a scheme to convert attenuation to temperature using the viscosity of the bcc phase of iron^15,25^, which is compatible with high attenuation, low shear-modulus, and high anisotropy (in contrast to the hcp phase of iron). Viscosity and attenuation are connected by a linear relationship following the Stokes formula for the low-viscosity material (see section “Methods”), using an average temperature of 6000 K. The application of the Stokes formula is justified for the bcc iron phase, which behaves like a very viscous fluid^15^ while remaining a highly anisotropic crystal^47^.

If we retain the most extreme values for Q as they are reported in the attenuation study^41^, we obtain an IC surface temperature map with two hot regions: the first one is smaller and hotter, about 600 K above the average temperature, and centred on the northern part of South America (Fig. 4). The second is more extensive and does not exceed 300 K above the average temperature. It encompasses the southern part of Asia, from the Mediterranean in the west to Australasia in the east. The coldest region is sandwiched between these two hot provinces beneath the northern Atlantic Ocean, with temperatures about 200 K below the average. According to our map, the sharpest boundary exists between the hot upwelling along the northeast coast of South America and cool downwelling in the Atlantic. We also explore an alternative approach in which, rather than using the results of first-principles molecular dynamics, we employ the bulk modulus and viscosity values from PREM^11^. This approach yields similar or smaller IC surface temperature variations, depending on the Anderson-Grüneisen parameter used. However, if we discard the most extreme input Q values, based on the uncertainty in measuring t*^41^, we obtain two-times-smaller variation in temperature (− 100 to 300 K).

Upon a visual comparison of compressional-wave speed and attenuation quality-factor tomograms, a positive correlation between speed and attenuation (1/Q) is apparent, i.e., high (low) speed corresponds to high (low) attenuation. Such a positive correlation between seismic speeds and anisotropy has been observed previously for the IC’s upper part (although not for the outermost 100 km) on the global scale in the context of velocity and attenuation anisotropy^48^. Evidence for azimuthal anisotropy (as opposed to cylindrical) would be an observation of dependence of differential travel-time residuals on the ray bottoming-point azimuth φ, measured relative to the north. For example, if low-order convection is frozen near the IC surface, we might expect higher differential travel time residuals near 0° or 180°^48^. The observation of a radial transverse anisotropy might also be consistent with an ancient convecting region in the upper part of the IC.

## Discussion

Suppose our tomographic images are interpreted as the thermal fingerprint of a convective circulation in the IC. Most likely, thermal and compositional convection provide a common mechanism; however, we concentrate on pure iron only, especially considering that the percentage of light elements in the inner core is low*.* In that case, we can estimate the flow pattern by computing the instantaneous response to the thermal buoyancy anomaly (see Fig. 4B and supplementary material for computation details). We expect a positive correlation between high attenuation and high compressional-wave speed due to the overlap between the degree of LPO that develops and the intrinsic attenuating nature of Fe-bcc. However, the strength of LPO varies within the convection cell, and its variation is likely responsible for the observed degree of seismic anisotropy. The upwelling “plume” material is seen to flow radially away at the surface in the thermal boundary layer, where we expect high shear-strain rates. The iron crystals are also likely to be oriented radially away from the hot spot along the IC surface, as shown in Fig. 4B. A seismic signal propagating along the surface will thus be averaged over all those directions, leading to a path-averaged velocity similar to the reference model.

We systematically analyze anisotropy on a global scale and concentrate on three distinct regions with good azimuthal coverage (see section “Methods”, and Figs. S10–S12). We find that complex anisotropic features exist rather than simple dependencies of PKIKP velocity with azimuth, which supports higher-order thermal convection. The maximum measured anisotropy is 2%, and a combination of crystal orientation and stress field can explain the observed pattern. An apparent positive correlation between the high velocity and high temperature could result from the fast flow of the hot upwelling material that can develop a high degree of LPO, thus increasing seismic velocity.

For IC viscosity values of ${10}^{9}$ Pa.s or smaller, we expect that classical Rayleigh-Bénard convection cells will form preferentially to the translational convection mode, particularly if the deepest parts of the inner core become stagnant. If the bcc phase of iron with much lower viscosity is stabilized in the IC (note that there are no experimental estimates on the bcc iron conductivity though reasonable theoretical predictions exist^49^), then higher convection modes will exist, with classical plume-style convection (see section “Methods” for more details). We consider one or two orders of magnitude Rayleigh number at the lower end of the range, resulting in expected speeds at the surface of the IC that lie in the range 10^−4^–10^2^ m/s. See Fig. 4B to illustrate the flow lines and strain patterns for one of the convection models.

Our observations of lateral variations in the temperature in the inner core indicate a substantial departure from the 1D radial structure. The observations establish that a full, 3-D analysis of the thermal structure is necessary, and we do so in the context of the solid-state flow that is implied by the existence of a 3-D structure. We show that convective circulation can occur given the constraints of current information on material properties and energy bounds. The question that follows is whether there are additional heat sources that are sufficient to drive convection while satisfying the upper bound imposed by the overall energy budget of the core.

The answer to the above question is yes; there are internal sources of heating, both localized (phase transition and/or re-crystallization) and non-localized (unstable isotopes). If the innermost inner core (IMIC) is a transition between iron phases or a recrystallization boundary, it might present a mechanism that can release latent heat and drive convection in the IC. The heat sources within the IC are constrained by the heat budget of the outer core and mantle. Within the bounds of the uncertainty of the energy budget at the ICB (below 1 to 2 TW; see our discussion in the Methods section, in the subsection *Heat Flux Constraints*), there is sufficient energy to drive convective overturn. We argue that it is difficult to rule out IC convection based on simple estimates of the balance between heat generation and thermal conductivity^50^ and, instead, it is important to undertake modelling of thermal convection under IC conditions based on our new understanding of the physical properties of the IC.

Finally, our data is sensitive only to the outermost IC, although the thermal convection implies the IC surface’s connection with its deep interior. The most recent robust parameter search^42^, the evidence from coda correlation^51^ and the travel times of ricochet waves^52^ confirm the existence of IMIC about halfway to the IC center, with a distinct style of anisotropy. The IMIC has a much more pronounced difference between the slow and fast axes of anisotropy than the rest of the IC. The IMIC could consist of a set of oriented iron crystals, and the new data presented here provides a holistic picture of the IC. One plausible explanation is that the whole IC was convecting sometime in the past, and the lattice-preferred orientation developed. As the core cooled down, its innermost part stopped convecting and preserved the orientation of iron crystals, prominent in the seismological data. The upper part of the IC is still convecting, which explains the more complicated seismic anisotropy observed seismologically. The transition from IMIC to the rest of the IC manifests a transient state in the Earth’s history^53^ that left the IMIC’s high order of crystal alignment frozen while the rest of the IC is still in a slow-convection mode.

Provided that the assumption of thermal convection allows the seismic data to be described, the thermal convection is likely. The ultimate answer to the question of plausibility will be established through further progress in developing more adequate models of the IC and progress in seismic studies and mineral physics.

## Methods

### PKiKP-PKIKP differential travel-time measurements

The most common methods to study the Earth’s inner core (IC) involve measuring travel times and amplitudes of seismic body waves that propagate through the Earth’s interior from the sources to the receivers and traverse the IC (Figs. 1, S1). Differential travel times between PKIKP waves that penetrate the depths of ~ 30–110 km beneath the IC boundary (ICB) (occur sooner in the seismograms) and PKiKP waves that reflect from the ICB (occur later in the seismograms) are measured using cross-correlation waveform matching as well as a handpicking and visual inspection to obtain the best fit between PKIKP and PKiKP waveforms. Here, we augment the past dataset^46^ with new measurements. We assemble 5477 differential travel times between PKIKP and PKiKP waves. The IC’s top-layer coverage by our data in a 3D view is shown in Fig. 1. The coverage and the differential travel-time residuals relative to the model ak135^53^ are plotted in a 2D view in Fig. S1. Due to seismic sources and receivers’ configuration, the eastern hemisphere is better sampled than the western hemisphere, and the northern hemisphere is better sampled than the southern hemisphere.

The processing details and example waveforms, including synthetics, can be found in the previous publications^38,44,46,62^.

Here, we provide several individual examples of PKIKP and PKiKP waveforms and include three supplementary Figs. S14–S16. Note that we also provide a supplementary text file with all events and stations used in the inversion.

### Method of IC compressional wave-speed tomography

Inversion for *Vp* perturbations to the outer 50 km of the IC (Fig. 2, S2–S3) utilizes the hierarchical Bayesian framework proposed recently^45,55^, described here with a slightly modified complexity selection criterion. *N* = 5477 pairs of short-period *PKIKP-PKiKP* teleseismic phase picks formed a dataset **d** of differential travel-time measurements, with peak sensitivity concentrated at the outermost IC and overlapping ray paths in the outer core, mantle, and crust suppressing source and receiver side structural effects outside of the region of interest. The sensitivity **G** of each differential ray-path pair to slowness perturbations **m** of each spherical harmonics up to degree *l′* was calculated by integration along the PKIKP path within the upper 50 km of the IC. We assume unknown Gaussian data noise σ_d_ and unknown slowness scale factor σ_m,_ both with half-normal hyperpriors with scales ${\nu }_{d}$ = 2 s and ${\nu }_{m}$ = 5 s/10^6^ m, respectively. To break the a priori correlation between the slowness scale σ_m_ and the slowness coefficients **m**, which improves sampling ability to traverse the posterior adequately, we adopt non-centered coordinates **m = **σ_m_ **m′**, with **m′** assumed to have a standard normal prior distribution. The log posterior is therefore given by

1 $$\begin{aligned} & {\text{log}}\left[ {p{(}\user2{m^{\prime}}, \sigma_{d} , \sigma_{m} {|}{\varvec{d}}} \right)] \propto - \frac{{\left| {\left| {{\varvec{G}}\left( {\sigma_{m} { }\user2{m^{\prime}}} \right) - {\varvec{d}}} \right|} \right|^{2} }}{{2\sigma_{d}^{2} }} + \frac{N}{2} {\text{log}}\left( {\sigma_{d} } \right) \\ & \quad + \frac{{\left( {l^{\prime } + 1} \right)^{2} }}{2}{\text{log}}\left( {\sigma_{m} } \right) - \frac{{{\varvec{m}}^{\prime 2} }}{2 } - \frac{{\sigma_{d}^{2} }}{{2 \nu_{d}^{2} }} - \frac{{\sigma_{m}^{2} }}{{2 \nu_{m}^{2} }} \\ \end{aligned}$$

We draw samples from the posterior using the highly efficient Hamiltonian Monte Carlo (HMC) method^56,57^, implemented using the STAN software package^58^, utilizing six chains of 1000 burn-in and 1000 retained samples each for the final run, for a total of 6000 posterior samples. The chains were compared to ensure convergence and correctness using standard HMC performance metrics.

To determine the best tradeoff between model complexity and the available data (i.e., to choose the optimal *l′* for the inversion), we employed a cross-validation strategy. Even when using differential travel time data, errors in the data likely manifest as correlations between subsets of the data sharing a source earthquake or receiver station. As such, we chose a conservative strategy of performing fivefold grouped cross-validation, with the source earthquake for each datum providing the grouping index, to evaluate the posterior predictive performance for *l′* = 1–13. The cross-validation score is given by the log expectation of the posterior predictive density as calculated by MCMC

2 $$CV - Score\left( l \right) = \mathop \sum \limits_{i}^{{N_{folds} }} \log {\mathbb{E}}\left[ {p{(}{\varvec{d}}_{{out\user2{ }}} {|}{\varvec{d}}_{in} , l)} \right]$$

where ${{\varvec{d}}}_{out}$ refers to the data held out of the current fold and ${{\varvec{d}}}_{in}$ is the data included in the current fold. For each fold, we used six chains with 500 burn-in and 500 retained samples each for the final run, for a total of 3000 posterior samples. This helps to minimize the impact of error due to source side effects, which for the data used in this study are less accounted for than receiver side effects. However, these effects are already minimal due to the ray path’s proximity. Applying Occam’s razor, we chose the lowest *l*′ for which the difference in posterior predictive performance did not significantly (> 1 standard error) improve for any higher *l*′, which for this data was *l*′ = 7, as seen in Fig. S8.

Additionally, Fig. S18 shows the residual distribution before the inversion and for the selected converged models.

### Synthetic tests for tomographic inversion

We design several synthetic experiments to test our Hamiltonian Monte Carlo tomographic inversion capacity^55–57^. We set up a forward problem that includes the ray tracing through the outermost IC using various shapes of velocity anomalies and ak135^54^ as a reference model. The first synthetic experiment (Fig. S6) shows that it is successful in recovering a hemispherical velocity-anomaly pattern, with a reasonably sharp boundary, except in the polar regions where the geometric coverage is low. In the second synthetic experiment (Fig. S7), we superimpose the elliptical shape of velocity anomaly, approximately South America’s size, onto the hemispherical pattern. Inside these elliptical anomalies, we add smaller-scale rhombi, roughly the size of Australia. The experiment reveals that it is possible to recover a multi-scale anomaly pattern, although the smallest scale cannot be recovered. Our synthetic test with a hemispherical boundary reveals that the boundary is recovered as sharp in the northern hemisphere, but becomes somewhat smoothed out in the southern. Overall, our experiment provides a fruitful result that gives confidence in resolving the anomaly patterns at the top of the IC.

We assume an ideally uniform inner core boundary so that the PKiKP phase can be used as a reference, and the relative travel time/amplitude variation is ascribed to the interior IC structures. However, we acknowledge that the method does not account for structures like IC mushy zones that were inferred seismologically^59^.

### Method of IC attenuation quality-factor tomography

In Fig. 3 (also see Figs. S4–S5), we plot the attenuation quality-factor tomogram and its uncertainty using the same color scale. This tomogram was derived using an independent dataset of PKIKP and PKPbc waves, sensitive to the IC’s outermost 400 km thick layer, and a method described in detail in a previous study^41^. A Bayesian trans-dimensional scheme using spherical Voronoi cells was employed as an inversion strategy. A similarity with the inversion for compressional-wave speed (Fig. 2) is that we treated the number of free parameters in the model as a free parameter itself and avoided explicit regularization.

### Method of measuring azimuthal anisotropy in the outermost inner core

Our PKiKP-PKIKP differential travel-time dataset is sensitive to the IC exterior, defined as the IC’s upper, ~ 100 km-thick shell. It is the most appropriate dataset to study the outermost IC because of the IC penetrating PKIKP waves that sample that layer laterally (see Fig. 1) rather than radially as in PKPbc-PKIKP of PKPab-PKIKP differential travel time datasets. Many studies reported that the outermost IC layer is isotropic because a dependency of PKIKP travel times on the Earth’s rotation axis’ (ERA) angle could not be established for the PKPbc-PKIKP dataset. However, in such a thin shell relative to the IC radius, it is appropriate to search for azimuthal anisotropy by calculating each PKIKP ray’s azimuth in its bottoming point instead of the angle relative to ERA. If the IC convection is sufficiently slow, it will form a lattice-preferred orientation (LPO) of crystals in the direction of flow in the upper part of the convection cells—flat sheets of the convection material parallel to the IC surface. The observation of a radial anisotropy might also be consistent with an ancient convecting region in the upper part of the IC. For a low Rayleigh number, the flow will follow the meridians, but this will no longer be the case for higher Rayleigh numbers. If anisotropy exists, it will manifest itself via faster propagation along the flow lines. However, as the flow lines diverge, especially near the upwelling or downwelling, this will make the interpretation difficult because there will be no clear dependence of travel time residuals on azimuth unless there is a large number of ray paths crossing a small region over the entire range of azimuths. Instead, we might expect to see clusters of travel-time residuals displaying a significant spread.

We can use the estimated temperature field (Fig. 4A) as a proxy of the flow-line complexity using the steepest gradient from hot to cold regions (Fig. 4B). We should keep in mind that more complex flow patterns can develop, which is currently beyond the resolution of our data. Anisotropy will manifest itself as a regional directional variation, depending on the geometry of flow. In the absence of heterogeneity, the maximum measured difference in the observed travel times of the rays sampling a region of interest will be a proxy for anisotropy’s strength that can develop. Such regional variation in both velocity and attenuation, with a positive correlation between them, has been reported for the IC’s African region^60^ and interpreted utilizing oriented anisotropic crystals found in laboratory experiments^61^.

Motivated by the previous studies of the relationship between compressional-wave speed and attenuation^48,60^, we investigated three IC regions where there exists a relatively good azimuthal coverage of PKiKP-PKIKP differential travel times: Africa, southern Pacific, and eastern Asia. These three regions are outlined in Fig. S1, which also shows the segments and the direction of PKIKP ray paths sampling the IC. Differential travel time residuals are shown as a function of the PKIKP midpoint azimuth for three different depths of IC outermost layer’s sampling: H < 30 km (top), H = 30–60 km (middle), and H > 60 km (bottom).

Beneath Africa (Fig. S10), we find evidence for variations in travel times, with the slowest paths for the azimuths of 45–50° and fastest for the azimuths of 25–30°. However, the travel time residuals show significant scatter and cluster in specific azimuthal ranges rather than distribute uniformly over a range of azimuths. They sample the IC beneath Africa in two distinct regions according to two different scenarios: (a) the quasi-western (slower) and quasi-eastern (faster) hemispheres, and were used to define the so-called hemispherical boundary^62,63^; (b) the two different domains, the Atlantic downwelling in the west (slower) and the Asian upwelling in the east (faster), and define a domain boundary as in this study (Fig. 4). The maximum difference in differential travel times for the rays sampling intermediate depths is about 1.5 s. For a PKIKP ray path bottoming 60 km beneath the ICB (spending ~ 66 s in the IC), this corresponds to about anisotropy’s 2.2% strength.

The second region we test is the south Pacific, centered at the boundary between the quasi-eastern and quasi-western hemispheres in more orthodox, hemispherical models of the IC. However, this region also represents a domain boundary between the south Pacific downwelling in the east and east Asia upwelling in the west (Fig. 4). We find a similar range of differential travel time residuals (Fig. S11) for the Africa region, resulting in anisotropy strength > 2%, although the azimuth ranges at which we measure the fastest and the slowest PKIKP waves are somewhat different. Finally, we analyze the east Asia region (Fig. S12), away from the domain boundaries, in the middle of upwelling in our model. Although we find a similar scatter in differential travel time residuals, we do not observe data clusters and cannot establish any dependence of speed on azimuth. Indeed, if the flow is divergent around the upwelling, we expect P waves’ fast propagation in all directions.

On a global scale, for low order convection, we would expect the fastest direction of PKIKP waves aligned with 0 or 180°, or if there is a significant dependency on azimuth at different angles, this could be either due to a combination of crystal orientation and the stress field^64^ or the effect of Earth’s rotation^60^. However, we do not find evidence for a systematic dependency of PKiKP-PKIKP differential travel-time residuals on azimuth, shown in Fig. S13. Thus, our result supports higher-order convection, in which anisotropy can be estimated only in terms of its absolute strength. The fact that we observe it means that the flow is likely slow enough for the crystal alignment to develop. We estimate the flow velocity by comparing the IC and outer core’s viscosity to be between 0.3 and 300 m/yr.

### Method of computing temperature from the quality factor

The quality factor is defined as follows

3 $$Q = 2\pi \left( {\frac{E}{\delta E}} \right),$$

where *E* is the energy of the sound wave and *δE* is the energy change (small compared to *E*) after the wave propagates through the material. The energy of the seismic wave is related to its amplitude, and the relationship between *Q* and the amplitude change can be written as^65^

4 $${{A_{final} } \mathord{\left/ {\vphantom {{A_{final} } {A_{initial} }}} \right. \kern-0pt} {A_{initial} }} = W\,exp\left( { - \pi fd/VQ} \right),$$

where *A*_*final*_ and *A*_*initial*_ are amplitudes of the soundwave after and before traveling through the material with thickness *d*, *f* is the frequency of the wave, *W* is the normalization term that depends on the geometry of the problem and is reversely proportional to the reflection coefficient, and the geometrical spreading factor. Finally, *V* is the velocity of sound waves.

5 $$ln\left( {A_{final} /A_{initial} } \right) = - \pi fd/VQ \, + ln\,W$$

The normalization term, *W*, is related to geometry so that it becomes close to 1 when the reflection coefficient and geometric spreading are very small, and it becomes close to 0 when R and G are large. In the case of homogeneous material where dissipation is large and *W* close to 1, the *ln(W)* is near 0 and likely much smaller than the – *πfd/VQ* term, and, therefore

6 $$ln\left( {A_{final} /A_{initial} } \right) = - \pi fd/VQ$$

The approximation above holds for the PKIKP waves sampling the upper layer of the IC used to derive Q tomograms^41^. Using the slowness of PKiKP waves and the values determined in a previous study (^66^; see their Fig. 1b), PKIKP waves at 146° correspond to the incidence angle of 46°, while those at 156° to the incidence angle of about 34°. Therefore, for the lower end of the PKIKP epicentral distance range (i.e., 146°), the reflection coefficient at the ICB is almost 0. For the upper end (i.e., 156°), it increases to about 0.02 (2%). The geometric spreading implies that the amplitude of the PKIKP decreases with distance, and for large dissipation (small Q) in the IC, this effect is small.

The IC’s material viscosity assuming the body-centered cubic (bcc) iron alloyed with some Ni and a low concentration of light elements^25^, is close to that of a very viscous liquid^15^. Therefore, the attenuation in the inner core can be expressed via wave amplitudes using Stokes formula^67^

7 $$A_{final} = A_{initial} exp\left( { - \alpha d} \right)$$

where

8 $$\alpha = {{2\eta \omega^{2} } \mathord{\left/ {\vphantom {{2\eta \omega^{2} } {\left( {3\rho V^{3} } \right)}}} \right. \kern-0pt} {\left( {3\rho V^{3} } \right)}}$$

*ƞ*—viscosity, *ω*—sound angular frequency,* ρ*—density, and *V*—sound speed in the medium. From Eq. (S7), we get

9 $$ln\left( {A_{final} /A_{initial} } \right) = - \alpha d$$

From this and Eq. (S6), we obtain that

10 $$- \alpha d = - \pi fd/VQ$$

and finally,

11 $$\eta Q = 3\rho V^{2} /8\pi f$$

The viscosity *ƞ*

12 $$\eta = \eta_{0} exp\left( { - \alpha T} \right)$$

is exponentially dependent on temperature with *ƞ*_0_ = 1.145 × 10^10^ Pa·s and α = 2.996 × 10^–3^ K^−1^ while density *ρ* and speed of sound *V* weakly depend on temperature^25^. Besides, the velocity is inversely proportional to the square root from density; therefore, neglecting the temperature dependence of the right-hand side in Eq. (S11) is well justified. Thus, for the given frequency *f,* the right-hand side of Eq. (S11) can be considered constant in the narrow range of temperatures.

13 $$\eta Q = A_{IC}$$

This constant *A*_*IC*_ can be obtained by assuming an average temperature at the surface of the IC. Namely, from the calculation of the average *Q* and viscosity near the IC surface, the constant is obtained as

14 $$A_{ic} = Q_{aver} \eta_{Tsurface}$$

Subsequently, for any given *Q* we can compute the corresponding ƞ (Eq. S13) and obtain the temperature as

15 $$T = ln\left( {\eta_{0} /\eta } \right)/\alpha$$

The *Q*_*aver*_ = 374.1. Assuming the *T*_*surface*_ = 6000 K, we obtain *ƞ*_*Tsurface*_ = 178.9 Pa·s. The viscosity changes from 336 to 30 Pa·s between 5800 and 6600 K (the range of surface temperatures assuming the average temperature of 6000 K). The assumption of the lower and higher average surface temperatures (e.g., 5500 and 6500 K) does not appreciably change the temperature variation. The distribution of temperatures on the IC surface remains the same, as shown in Fig. 4A (see also Fig. S9). Finally, we note that if the IC material’s viscosity is in the range of 10^18^ Pa s, then the material would behave as almost ideally elastic and result in nearly zero attenuation.

### Alternative method of computing temperature

A plausible alternative approach to estimate IC temperature and viscosity would be starting from Eq. (S11), where *ρV*^*2*^ is *K*_*S*_, the adiabatic bulk modulus, under the same assumption used about the Stokes relation. Measurements of the *K*_*S*_ of minerals led to the definition of the Anderson-Grüneisen parameter:

16 $$\delta_{S} = - \left( {\alpha K_{S} } \right)^{ - 1} \frac{{dK_{S} }}{dT} = - \alpha^{ - 1} d\,{\text{log}}\frac{{K_{S} }}{dT}$$

Specifically, assuming ${\delta }_{S}$ is constant,

17 $$K_{S} = K_{S0} {\text{exp}}\left[ { - \delta_{S} \mathop \smallint \limits_{{T_{0} }}^{T} \alpha \left( \tau \right)d\tau } \right]$$

Rewriting Eq. (S12) to emphasize *ƞ*’s dependence on changes from the reference temperature *T*_*0*_,

18 $$\eta = \eta_{0} {\text{exp}}\left[ { - \mathop \smallint \limits_{{T_{0} }}^{T} \alpha \left( \tau \right)d\tau } \right]$$

Rearranging Eq. (S11), we obtain

19 $$Q = \frac{{K_{S} }}{\eta } \frac{3}{8\pi f}$$

Finally, from Eqs. (S17) and (S18), and assuming constant $\alpha$, gives

20 $$Q = \frac{{K_{S} }}{{\eta_{0} }} \frac{{{\text{exp}}\left[ { - \delta_{S} \mathop \smallint \nolimits_{{T_{0} }}^{T} \alpha dT} \right]}}{{{\text{exp}}\left[ { - \mathop \smallint \nolimits_{{T_{0} }}^{T} \alpha dT} \right]}} \frac{3}{8\pi f}\user2{ } = \frac{{K_{S} }}{{\eta_{0} }} {\text{exp}}\left[ {\alpha \left( {1 - \delta_{S} } \right)\left( {T - T_{0} } \right)} \right]\frac{3}{8\pi f}$$

Our mean Q is ∼ 370 at reference conditions, and $\eta_{0}$ is $1.145 \times 10^{10} {\text{Pa}} \cdot {\text{s}}$. If the seismic wave frequency is 1 Hz, these values combine to provide a value for ${K}_{S0}$ of ∼ 35,615 GPa (from Eq. S11). The PREM bulk modulus of the IC is ∼ 1340 GPa.

Suppose we use the PREM value for the IC’s $K_{S0} .$ We get an $\eta_{0}$ of ∼ 430 ${\text{ MPa}} \cdot {\text{s}}$, about 27 times less than we used in Eq. (S12).

Let us define:

21 $$Q_{0} = \frac{{K_{S0} }}{{\eta_{0} }} \frac{3}{8\pi f}$$

and examine the variation of reported values of Q to estimate the temperature variation. Q ranges from a low of about 50 to a high of about 650. From Eq. (S20), we can derive:

22 $$ln\left( {\frac{Q}{{Q_{0} }}} \right) = \alpha \left( {1 - \delta_{S} } \right)\left( {T - T_{0} } \right)$$

Using our Q value range and $\alpha , \mathrm{we can}$ calculate the range $T-{T}_{0}$ as follows:

$$\begin{aligned} & \left[ {670\,{\text{ K}}, \, - 286\,{\text{ K}}} \right]\,{\text{ for}}\,\delta_{S} = 2 \\ & \left[ {223 \, \,{\text{K}}, \, - 62\, {\text{K}}} \right] \, \,{\text{for}}\,\delta_{S} = 4 \\ & \left[ {134\,{\text{ K}}, \, - 37} \right] \, \,{\text{for}}\,\delta_{S} = 6 \\ \end{aligned}$$

This is very similar to the range [600 K, − 200 K] shown in Fig. 4. In turn, those T variations, employing Eq. (S18), lead to viscosities $\eta$ of between $3.7 \times 10^{7} {\text{Pa}} \cdot {\text{s}}$ and $5.3 \times 10^{8} {\text{Pa}} \cdot {\text{s}}$.

If the surface temperature of the IC is very low, close to 5000 K, then both methods produce reasonably close estimates of viscosity.

### Method for analysis of flow in the inner core

#### Dynamic models

Our tomographic imaging provides evidence for a large-scale density variation in the IC. These variations have implications for the pattern of flow within the IC and for the heat flux into the outer core.

The two alternative models for converting seismic Q to temperature and viscosity imply reference viscosity values in the range ${10}^{3}$–${10}^{9}$ Pa·s, lateral viscosity variations of the order of 100–1000 Pa·s, and temperature variations of the order of 100 K.

Both the viscosity values and the temperature gradients are substantially more favorable for convective instablity compared to the values used in previous analyses^68,69^ that argued the conditions in the IC are below the critical value for self-sustaining convective circulation.

The planform of anomalies that we observe, if derived from convective circulation, is consistent with moderately super-critical convection with length-scales comparable to the depth of the convecting layer.

#### Heat flux constraints

There is no information on vertical gradients in temperature or viscosity in the tomograpic model, but the vertical temperature gradient is constrained by the maximum heat flow into the base of the outer core.

Reference^70^ estimate the heat flux at the base of the outer core to be less than 5 TW and is more likely to be closer to 2.2 TW. Any heat flux from convective overturn in the inner core must lie within the uncertainty in these numbers. For the purpose of this analysis, let us assume that an upper bound on the heat flux from the inner core is close to 1 TW.

This implies a temperature gradient near the surface of the inner core of that cannot be higher than 0.5 to 1 K/km and, in turn, a minimum boundary layer thickness close to 100 km.

A boundary layer thickness of 100 km implies a Nusselt number of between 10 and 20, assuming the depth of convection is comparable to the radius of the inner core. This is also consistent with moderately super-critical convective overturn.

The average thermal boundary layer thickness is inversely related to the heat flow. If the heat flow drops below a 0.25 TW, then the thermal boundary layer thickness is comparable to the thickness of the convecting layer and implies little or no convective overturn.

#### Mathematical formulation

In the following analysis, we develop a mathematical formulation to help determine whether the observed density variations are transient and decaying, or self-sustaining.

All materials, including crystalline solids, undergo irreversible deformation in response to stress even if that deformation occurs at very low strain rates. This means that over a long enough timescale, the equations of fluid dynamics apply and we can determine an effective viscosity for the solid material.

The Navier–Stokes equation describes the momentum balance in a viscous fluid in response to buoyancy forces and pressure gradients. In a rotating, conducting material such as the inner core in the presence of the external magnetic field from the geodynamo, we may also need to consider the influence of Coriolis, centrifugal and Lorentz forces^71,72^.

23 $$\uprho \frac{\partial u}{{\partial t}} +\uprho \left( {u \cdot \nabla } \right)u + 2\uprho \Omega \times u +\uprho \Omega \times \left( {\Omega \times x} \right) - \nabla \cdot \left[ \upeta \left( {\nabla u + \nabla u^{T} } \right) \right] + \nabla p =\uprho g + \frac{1}{{\mu_{0} }}\nabla \times B \times B$$

In this equation, $\uprho$ is the density, $u$ is the velocity, $\Omega$ is the rotation rate vector, $\upeta$ is viscosity, $p$ is pressure, $g$ is the gravitational acceleration vector, ${\upmu }_{0}$ is the magnetic permeability of the vacuum, and $B$ is the magnetic field. The density, rotation rate and the electromagnetic contributions to this balance to have their own cross-coupled evolution equations but, as in any system where there are multiple different forces present, there are likely to be distinct regimes in which a subset of those forces dominate the balance, and others can be neglected. To understand the current system, we first non-dimensionalise using the inner-core radius, $d={r}_{ic}$ as the length scale, a thermal diffusion timescale, ${d}^{2}/$, temperature scaling from the expected temperature range, $\Delta T$, and a mass (stress) scaling based on a reference viscosity, ${\upeta }_{0}$ such that stresses or pressure scale as $p={\upeta }_{0}\upkappa /{d}^{2}{p}{\prime}$.

For simplicity, we replace the arbitrary-oriented $\Omega$ with ${\Omega }_{0}\widehat{z}$ and obtain the following non-dimensional expression

24 $$\frac{{\uprho _{0}\upkappa }}{{\upeta _{0} }}\left[ {\frac{{\partial u^{\prime } }}{{\partial^{\prime } }} + {\uprho }\left( {u^{\prime } \cdot \nabla^{\prime } } \right)u^{\prime } + 2{\Omega }_{0} \hat{z} \times u^{\prime } } \right] - \nabla^{\prime }\cdot \left[ \upeta ^{\prime } \left( {\nabla^{\prime } u^{\prime } + \nabla^{\prime } u^{\prime T} } \right)\right] + \nabla^{\prime } p^{{*^{\prime } }} = \frac{{g\uprho _{0} \upalpha {\Delta }Td^{3} }}{{\upkappa \upeta _{0} }}g + \frac{{B_{0}^{2} d^{2} }}{{\upmu _{0} \upkappa \upeta _{0} }}\nabla^{\prime } \times B^{\prime } \times B^{\prime }$$

In this expression dimensionless variables and derivatives are denoted using primes (e.g. ${x}{\prime}$). We have assumed the Boussinesq approximation for the relation between density and temperature. Pressure-like terms are all gathered into ${p}^{*}$ and include contributions from the centrifugal force term and the Lorentz force (see for details of the scaling).

There are four non-dimensional numbers that appear in this analysis.

25 $$\frac{1}{Pr} = \frac{{{\uprho }_{0} {\upkappa }}}{{{\upeta }_{0} }};\quad {\Omega }^{*} = \frac{{{\Omega }_{0} }}{Pr}$$

The Prandtl number (${\text{Pr}}$, appearing as its inverse) scales thermal to viscous diffusion, and informs us of the importance of inertial effects. The effect of rotation is given by ${\Omega }^{*}$ which is the angular velocity scaled by Prandtl number.

The thermal Rayleigh number:

26 $$Ra = \frac{{g\uprho _{0} \upalpha \Delta Td^{3} }}{{\upkappa \upeta _{0} }}$$

is a measure of the importance of thermal buoyancy forces to the other force terms. $\mathrm{\alpha }$ is the thermal expansivity We acknowledge that this is a simplification since we expect compositional variations also to contribute to buoyancy. The final dimensionless number determines the relative importance of the Lorentz force

27 $$L = \frac{{B_{0}^{2} d^{2} }}{{\upmu _{0} \upkappa \upeta _{0} }}$$

This is equivalent to the Elasasser number divided by the magnetic Prandtl number and is interpreted as the ratio of the Joule damping timescale to the stress diffusion timescale.

Dropping primes for convenience, we can now write the dimensionless form of the Navier–Stokes equation as

28 $$\frac{1}{\Pr }\left[ {\frac{\partial u}{{\partial t}} + u \cdot \nabla u} \right] + \Omega^{*} \hat{z} \times u - \nabla \cdot \left[ \upeta \left( {\nabla u + \nabla u^{T} } \right)\right] + \nabla p^{{ \star }} = RaT + L\nabla \times B \times B$$

Our range of viscosity estimates implies a Prandtl number between $O\left({10}^{4}\right)$ and $O\left({10}^{10}\right)$ which is in the creeping flow regime (inertia does not play a role in the dynamics of the system). The value of ${\Omega }^{*}$ is estimated to be $O\left(1\right)$ to $O\left(0.01\right)$ for flow confined to the shallow inner-core but may become $\gg 1$ if the flow depth extends across the entire inner core and the viscosity is at the lower end of the estimated range. In this case, Coriolis forces need to be considered. The parameter $\upeta$ in the equation.

The Rayleigh number is estimated to be $O\left({10}^{23}\right)-O\left({10}^{9}\right)$ for the range of viscosity and length scales that we need to consider. The Lorentz term is between $O\left({10}^{14}\right)-O\left({10}^{6}\right)$ for this same range of parameters and is therefore always significantly smaller than the buoyancy effect. We have assumed that the value of ${B}_{0}$ is similar to that of the outer-core field of ${10}^{-3} T$ although it has also been suggested that a higher value near the ICB is possible if we consider the toroidal field. This is still significantly smaller than the thermal buoyancy contribution.

The remaining terms describe a steady state balance between buoyancy forces and viscous retarding forces in the presence of Coriolis forces. The density (temperature) variations that are obtained from our attenuation tomography imply both the flow pattern and intensity.

29 $$\Omega^{*} \hat{z} \times u - \nabla \cdot \left[ \upeta \left( {\nabla u + \nabla u^{T} } \right) \right] + \nabla p^{{ \star }} = {\text{Ra}}T\left( {r,\phi ,\theta } \right)$$

This solution is time independent. The flow should be well-defined by knowing $Ra$ and ${\Omega }^{*}$. The viscosity.

#### Computational formulation and model

We implement our model using the Underworld finite element code by Ref.^73,74^, which makes extensive use of the PETSc computational framework for efficient parallel computation^75^. Underworld has a python interface and all the relevant scripts are made available.

The model consists of a spherical ball divided into an unstructured tetrahedral mesh of quadratic velocity, linear pressure elements with a free slip upper boundary and with a buoyancy force defined using the predicted temperature anomaly from the tomographic model. Since this model does not have depth information, we make the following assumption:

30 $$T\left(r,\upphi ,\uptheta \right)={T}_{\text{TM}}\left(\upphi ,\uptheta \right)\cdot {r}^{2}{\text{sin}}\left(\uppi {r}^{\prime}\right)$$

where *r*′ is the normalised distance from the centre of the IC ($${r/r}_{IC})$$

We assume that the relationship between temperature and viscosity is the same one that was used to convert attenuation to temperature.

31 $${\upeta }\left( T \right) = {\upeta }_{0} \exp \left( { - {\upalpha }_{0} T} \right)$$

We further assume that the variation of gravity with depth is linear

32 $$g = g \cdot r^{\prime}$$

#### Flow speed scaling

The surface flow pattern is illustrated in Figs. 4B, S14. The flow directions do not vary significantly with the viscosity structure but the influence of rotation does change the flow pattern significantly with the equatorial upwelling balanced by a polar downwellings. The magnitude of the flow speed that we obtain with these model scales as

33 $$V={V}_{0}\frac{\upkappa }{d}{\text{Ra}}$$

Provided the viscosity is Newtonian (linear with respect to strain-rate) and the Prandtl number is much larger than 1, ${V}_{0}$ is a purely geometrical factor that depends on the distribution of buoyancy relative to the boundaries, the variation of with depth, and the pattern of viscosity variations. We determine ${V}_{0}$ as the typical dimensionless flow speed in the numerical models (Table S1). The value of ${V}_{0}$ for the flow near the surface of the IC is ${10}^{-4}$ with only small changes that result if the viscosity jumps significantly at depth. Coriolis forces, if important, change the velocity pattern with significantly more flow close to the poles, but the scaling is not significantly changed. The interior velocity is more sensitive to the deep viscosity structure. Figure 4 shows the deformation of small strain markers seeded at depth in the flow pattern. When a stagnant innermost-inner-core shell (IMIC) is present, the strain that accumulates during a single convective overturn is substantially higher than in the absence of the IMIC. This is due to the significant (passive in our model) shearing of the flow close to the IMIC interface.

There is considerable uncertainty for most of the IC constitutive parameters including thermal expansivity, thermal diffusivity, and viscosity with the last of these being most poorly understood. The range of values we have assumed in the discussion above are an inner core density of $13000$ kg/m^3^, thermal conductivity of $100-300$ W/m/K, heat capacity of $650$ J/kg/K, and thermal expansion coefficient of $10-30\times {10}^{-6}/{\text{K}}$, the range of thermal diffusivity is $12-36\times {10}^{-6} {{\text{m}}}^{2}/{\text{s}}$, and a reference viscosity in the range ${10}^{3}$–${10}^{9}$ Pa·s.

For the upper end of the viscosity estimates, the Rayleigh number falls into the range ~ 10^8^–10^9^, and the effective Rayleigh number at the surface of the IC is ~ 10^5^–10^6^ due to higher viscosity in the boundary layer. This is in the range where we expect classical Rayleigh-Bénard flow patterns with a single characteristic length scale.

If the reference viscosity is lower, then the predicted Rayleigh number is up to $\sim {10}^{12}$, and the Reynolds number could be $\gtrsim 100$ above which steady, laminar flow begins to break down and we approach the onset of turbulent flow. This is not consistent with the anomalies we observe having a convective origin and so limits the range of viscosity to 10^6^–10^9^.

Recall that the temperature range is an upper bound under the assumption that the viscosity variations are purely thermal. It is not unreasonable to consider at least one or two orders of magnitude Rayleigh number at the lower end of the range, resulting in expected speeds close to the surface of the inner core that lie between 10^−4^ and 10^2^ m/s.

## Discussion

The viscosity variations implied from the attenuation model are 2–3 orders of magnitude in the uppermost few tens of km of the inner core. This suggests that our models in the lower Ra range will fall into the “sluggish lid” convection regime in which boundary layer thickness, surface flow speeds, and heat transport across the boundary layer are strongly sensitive to the thermal dependence of the viscosity. We cannot rule out the possibility that a somewhat higher temperature sensitivity would imply that the models are in a stagnant-lid regime with boundary layer geometry determined by rheology rather than temperature. In the absence of depth sensitivity in the tomographic models, the planform of the flow is the most robust component of our analysis.

### Supplementary Information

Supplementary Information.

## Data Availability

The data that support the findings of this study are available from the corresponding author upon reasonable request.
