# Supplementary material for: Imaging the top of the Earth’s inner core: a present-day flow model
Source: Sci Rep. 2024 Apr 18;14:8999. doi: 10.1038/s41598-024-59520-7 (PMC11026418; doi:10.1038/s41598-024-59520-7)
Supplement: Supplementary file 1 — Supplementary Information. [file 41598_2024_59520_MOESM1_ESM.docx]

**Supporting Information for**

Imaging the top of the Earth’s inner core: A present-day flow model

Hrvoje Tkalčić^1^, Anatoly B. Belonoshko^2^, Jack B. Muir^3^, Maurizio Mattesini^4,5^, Louis Moresi^1^ and Lauren Waszek^6,7^

**Affiliations:**

^1^ Research School of Earth Sciences, The Australian National University, Canberra, ACT 2601, Australia

^2^ Department of Physics, Royal Institute of Technology, SE-100 44, Stockholm, Sweden

^3^ Department of Earth Sciences, University of Oxford, Oxford, OX1 3AN, UK

^4^ Department of Earth’s Physics and Astrophysics, Complutense University of Madrid, Madrid, Spain.

^5^ Instituto de Geociencias (UCM-CSIC), Facultad de Ciencias Físicas, Madrid, Spain

^6^ Physical Sciences, James Cook University, Townsville, QLD 4810, Australia

^7^ Department of Physics, New Mexico State University, Las Cruces, NM 88003, USA

* Corresponding author. Email: Hrvoje.Tkalcic@anu.edu.au

**This PDF file includes:**

Figures S1 to S18

Table S1

Legends for Movie S1

**Other supporting materials for this manuscript include the following:**

Movie S1

Fig. S1.

Ray-path geometry and amplitude of travel-time residuals sensitive to the inner core (IC). Left column: Surface projections of PKIKP ray-path segments in the IC. Three regions are outlined for which a detailed analysis of azimuthal anisotropy is performed: the Africa region in orange (see fig. S10), the South Pacific region in green (see fig. S11), and the East Asia region in cyan (see fig. S12) centered on 0° (top) and 180° (bottom). Right column: Same, but PKiKP-PKIKP differential travel time residuals with respect to the model ak135 (*52*) are plotted at their bottoming points in the IC.

Fig. S2.

Tomograms of the compressional speed in the outermost IC. The same values are plotted as in Fig. 2 (left column), but using a different projection. The image in the middle shows the model centered on the equator and the zeroth meridian. The images to its left and right are centered on the longitudes of 270°, 90°, and 180°, respectively. The images above and below are centered on the latitude of 90° and −90°, respectively.

Fig. S3.

The standard deviation of the compressional velocity tomogram in the outermost IC. The same values are plotted as in Fig. 2 (right column), but using a different projection. The image in the middle shows the standard deviation map centered on the equator and the zeroth meridian. The images to its left and right are centered on the longitude of 270°, 90°, and 180°, respectively. The images above and below are centered on the latitude of 90° and −90°, respectively.

Fig. S4.

Tomograms of the attenuation quality-factor in the outermost IC. The same values are plotted as in Fig. 3 (left column), but using a different projection. The image in the middle shows the model centered on the equator and the zeroth meridian. The images to its left and right are centered on the longitude of 270°, 90°, and 180°, respectively. The images above and below are centered on the latitude of 90° and −90°, respectively.

Fig. S5.

The standard deviation of the attenuation quality-factor tomogram in the outermost IC. The same values are plotted as in Fig. 3 (right column), but using a different projection. The image in the middle shows the standard deviation map centered on the equator and the zeroth meridian. The images to its left and right are centered on the longitude of 270°, 90°, and 180°, respectively. The images above and below are centered on the latitude of 90° and −90°, respectively.

Fig. S6.

Tomographic synthetic (recovery) experiment #1. Top: input model. Middle: the mean of the recovered compressional-wave speed model. Bottom: The standard deviation of the recovered compressional-wave speed model. Centered on the zeroth meridian (left) and the Pacific (right).

Fig. S7.

Tomographic synthetic (recovery) experiment #2. Top: input model. Middle: the mean of the recovered compressional-wave speed model. Bottom: The standard deviation of the recovered compressional-wave speed model. Centered on the zeroth meridian (left) and the Pacific (right).

Fig. S8.

Cross-validation method results. The difference in 5-fold grouped cross-validation (CV) performance, summed across folds, relative to degree *l'* = 7. Higher is better, and the standard error in the difference relative to *l'* is shown by the error bars. No degree higher than l’=7 has a significant improvement in CV predictive performance, so we chose l’=7 for our final inversion by application of Occam's razor.

Fig. S9.

Map of temperature field in the outermost IC. The same values are plotted as in Fig. 4A, but using a different projection. The image in the middle shows the map centered on the equator and the zeroth meridian. The images to its left and right are centered on the longitude of 270°, 90°, and 180°, respectively. The images above and below are centered on the latitude of 90° and −90°, respectively.

Fig. S10.

Azimuthal anisotropy analysis for the African region. PKiKP-PKIKP differential travel time residuals relative to PKIKP rays’ azimuth in their bottoming point’s in the IC at different depths: (top) 0-30 km, (middle) 30-60 km, and (bottom) deeper than 60 km. Data for the African region as defined in fig. S1 are shown in orange.

Fig. S11.

Azimuthal anisotropy analysis for the South Pacific region. PKiKP-PKIKP differential travel time residuals relative to PKIKP rays’ azimuth in their bottoming point’s in the IC at different depths: (top) 0-30 km, (middle) 30-60 km, and (bottom) deeper than 60 km. Data for the South Pacific region as defined in fig. S1 are shown in green.

Fig. S12.

Azimuthal anisotropy analysis for the African region. PKiKP-PKIKP differential travel time residuals relative to PKIKP rays’ azimuth in their bottoming point’s in the IC at different depths: (top) 0-30 km, (middle) 30-60 km, and (bottom) deeper than 60 km. Data for the East Asia region as defined in fig. S1 are shown in cyan.

Fig. S13.

Azimuthal anisotropy analysis. Rose histograms of the PKiKP-PKIKP differential travel time residuals relative to PKIKP rays’ azimuths in their bottoming points in the IC: (left) positive; (right) negative residuals. The residual magnitude is on the radial component.

Fig. S14.

Example waveforms from a selection of events across the dataset. PKIKP and PKiKP phases are indicated. Event and station code information are as follows, reading from top left downwards then top right downwards: 11-08-2007, Fiji, LSZ; 24-09-2010, Peru, AS31; 15-01-2015, Indonesia, MVL; 04-09-2011, Vanuatu, OJC; 07-07-2012, South Sandwich Islands, BRVK; 16-10-2012, Kuril, LCO; 08-06-2013, Argentina, KURK; 23-07-2014, Chile, PSAA1; 01-02-2015, Santa Cruz, VTS; 24-05-2015, South Sandwich Islands, OTUK; 01-09-2016, Sumatra, BGNE.

Fig. S15.

The same data as in S15, with the PKIKP waveform superimposed on the PKiKP waveform in grey.

Fig. S16.

Example waveforms from an individual event in Sumatra on 17-09-2016, showing the PKIKP and PKiKP as a function of event-receiver epicentral distance. Station codes are (in order of increasing epicentral distance): FXWY, ARV, LAO, REDW, SLA, DAN, RSSD, O20A, KAPO, F36B, ECSD, K31B, G40A, I37B, BGNE, N33B, Y22A, JFWS, VA03.

Fig. S17.

Residual histograms for PKiKP-PKIKP differential travel times before (black) and after (green) the selected P-wave velocity models for the top of the IC.


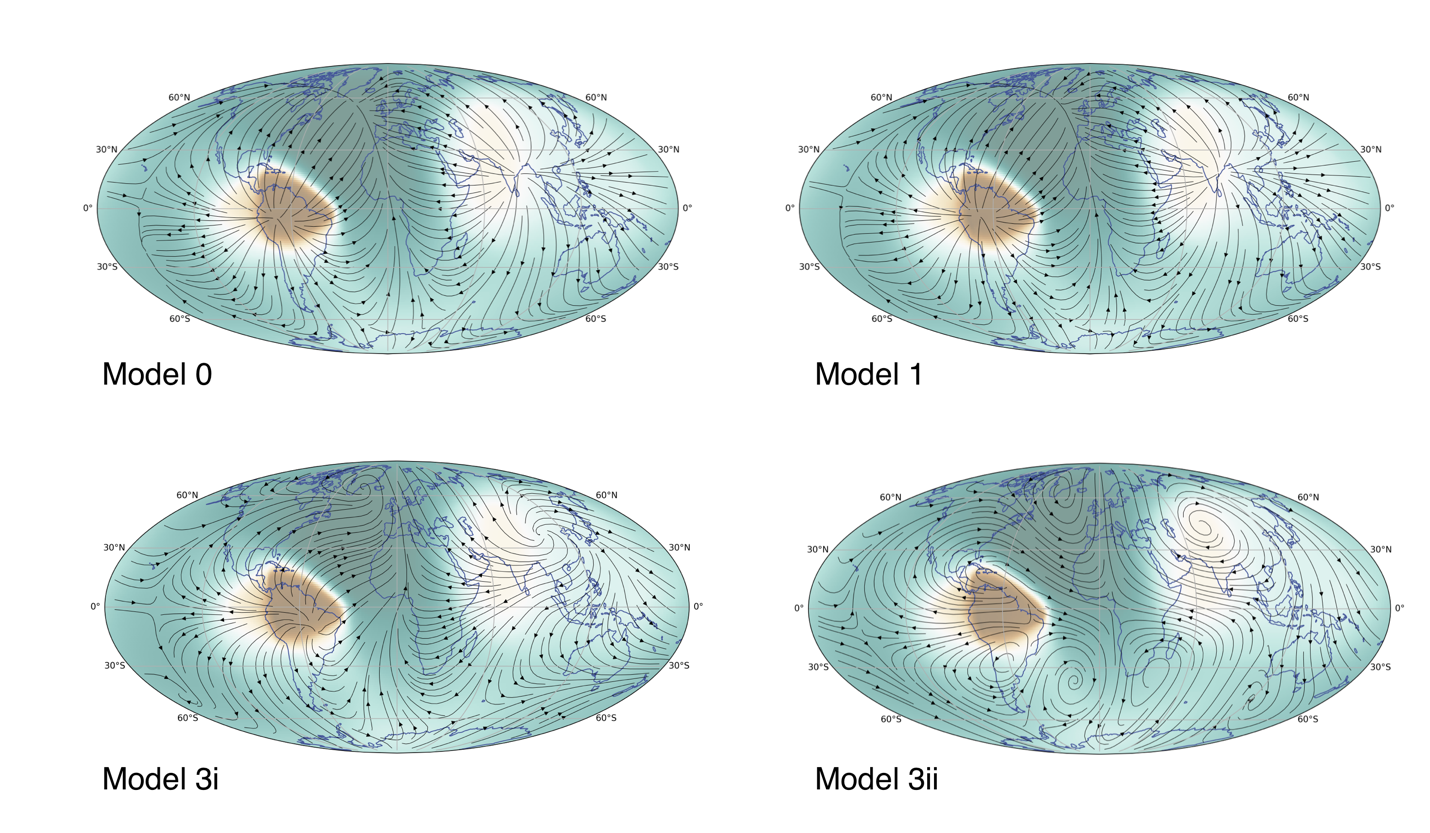


**Fig. S18.**

Flow directions at the upper surface of the IC. The flow models for four different cases from Table S1 span the end members. Surface flow structure and magnitude is very weakly sensitive to the deep viscosity but are strongly sensitive to the Coriolis force (Models 3.i and 3.ii in Table S1).

| Model | $C_{0}$ | $\eta(r)$ | $\eta_{i}/\eta_{0}$ | $\Omega^{*}$ | $V_{0}^{s}$ (surface) | $V_{0}^{i}$ (interior) |
| --- | --- | --- | --- | --- | --- | --- |
| 0 | 0.0 | 0.0 | 1 | 0 | $4.4\times{10}^{-4}$ | $5.5\times{10}^{-4}$ |
| 1 | $1\times{10}^{-2}$ | 0.0 | 1 | 0 | $6.0\times{10}^{-4}$ | $6.0\times{10}^{-4}$ |
| 2 | $1\times{10}^{-2}$ | Step @ 0.5 | 100 | 0 | $2.5\times{10}^{-4}$ | $3.1\times{10}^{-4}$ |
| 3 | $0$ | Step @ 0.5 | 100 | 0 | $1.7\times{10}^{-4}$ | $2.5\times{10}^{-4}$ |
| 3.i | 0 | Step @ 0.5 | 100 | 10 | $2.1\times{10}^{-4}$ | $3.0\times{10}^{-4}$ |
| 3.ii | 0 | Step @ 0.5 | 100 | 50 | $2.2\times{10}^{-4}$ | $3.0\times{10}^{-4}$ |

**Table S1**.

Flow model results. viscosity structure of each model run are parameterized via the constant, $C_{0}$ and a viscosity step at $r=0.5$ in models with an imposed high-viscosity inner core. The ratio viscosity at the innermost inner core to the reference value is $\eta_{i}/\eta_{0}$. $V_{0}$ is the geometrical factor that is required to scale velocity (equation 2). Different values are obtained for the models depending for the rms velocity at the surface ($V_{0}^{s}$) or the rms interior velocity ($V_{0}^{i}$).

Movie S1.

Left: 3D illustration of PKIKP ray-paths traversing the IC from a viewpoint outside the Earth. The Earth first spins eastward, and after a full rotation, it turns northward for another full rotation. The red globe in the center is the IC. The ray paths are from the PKiKP-PKIKP differential-travel-time dataset used in this study. Ray-path colors correspond to different values of differential-travel-time residuals: blue marks fast, white marks neutral, and red marks slow paths through the IC (with respect to the mean value). Green balls are station locations, and red balls are event locations. Right: Enlarged image of the IC, with the map of the Earth’s surface projected on the IC for orientation purposes. Yellow dots are the PKIKP piercing points. Animation credit: Stuart Ramsden, Vizlab, National Computational Infrastructure.
